# Pneumococcal conjugate vaccines and hospitalization of children for pneumonia: a time-series analysis, South Africa, 2006–2014

**DOI:** 10.2471/BLT.16.187849

**Published:** 2017-06-26

**Authors:** Alane Izu, Fatima Solomon, Susan A Nzenze, Azwifarwi Mudau, Elizabeth Zell, Katherine L O’Brien, Cynthia G Whitney, Jennifer Verani, Michelle Groome, Shabir A Madhi

**Affiliations:** a Medical Research Council: Respiratory and Meningeal Pathogens Research Unit, Faculty of Health Sciences, University of the Witwatersrand, York Road, Parktown, 2193, South Africa.; b National Research Foundation: SARCHI on Vaccine Preventable Diseases, University of the Witwatersrand, Johannesburg, South Africa.; c Stat-Epi Associates, Inc., Ponte Verde Beach, United States of America (USA).; d International Vaccine Access Center, Johns Hopkins Bloomberg School of Public Health, Baltimore, USA.; e Centers for Disease Control and Prevention, Atlanta, USA.

## Abstract

**Objective:**

To assess the impact of immunization with pneumococcal conjugate vaccines on all-cause pneumonia hospitalizations among children in Soweto, South Africa.

**Methods:**

We used data collected at the Chris Hani Baragwanath Hospital in Soweto between 2006 and 2014 – i.e. before and after April 2009, when a pneumococcal conjugate vaccine was first included in South Africa’s routine immunization programme. Using a Bayesian generalized seasonal autoregressive moving-average model and the data collected in 2006–2008, we estimated the numbers of children that would have been hospitalized for pneumonia between 2010 and 2014 if no pneumococcal conjugate vaccines had been used. These estimates were then compared with the corresponding numbers of hospitalizations observed.

**Findings:**

Between 2006 and 2014, 26 778 children younger than five years – including 3388 known to be infected with human immunodeficiency virus (HIV) – were admitted to the study hospital for pneumonia. We estimated that, for the children known to be infected with HIV and for the other children, pneumococcal conjugate vaccines reduced the numbers of hospitalizations for pneumonia in 2014 by 33% (50% credible interval, CrI: 6 to 52) and 39% (50% CrI: 24 to 50), respectively. In the study hospital in 2012–2014, as a result of immunizations with these vaccines, there were an estimated 3100 fewer pneumonia hospitalizations of children younger than five years.

**Conclusion:**

In our study hospital, following the introduction of pneumococcal conjugate vaccines into the national immunization programme, there were significant reductions in pneumonia hospitalizations among children.

## Résumé

**Objectif:**

Évaluer l'impact de l'immunisation à l'aide de vaccins antipneumococciques conjugués sur les hospitalisations d'enfants atteints de pneumonie, toutes causes confondues, à Soweto, en Afrique du Sud.

**Méthodes:**

Nous avons utilisé des données recueillies auprès de l'hôpital Chris Hani Baragwanath à Soweto entre 2006 et 2014, c'est-à-dire avant et après avril 2009, date à laquelle un vaccin antipneumococcique conjugué a été inclus pour la première fois dans le programme de vaccination systématique en l'Afrique du Sud. À l'aide d'un modèle bayésien généralisé saisonnier à moyenne mobile autorégressive et des données recueillies entre 2006 et 2008, nous avons estimé le nombre d'enfants qui auraient été hospitalisés pour une pneumonie entre 2010 et 2014 si aucun vaccin antipneumococcique conjugué n'avait été utilisé. Ces estimations ont ensuite été comparées au nombre d'hospitalisations observées correspondant.

**Résultats:**

Entre 2006 et 2014, 26 778 enfants âgés de moins de cinq ans – parmi lesquels 3388 dont la séropositivité au virus de l'immunodéficience humaine (VIH) était connue – ont été admis au sein de l'hôpital d'étude pour une pneumonie. Nous avons estimé qu'en 2014, les vaccins antipneumococciques conjugués avaient permis de réduire le nombre d'hospitalisations pour une pneumonie de 33% (intervalle de crédibilité, ICr, à 50%: 6-52) dans le cas des enfants dont la séropositivité au VIH était connue, et de 39% (ICr à 50%: 24-50) dans le cas des autres enfants. D'après nos estimations, le nombre d'hospitalisations pour une pneumonie d'enfants âgés de moins de cinq ans a diminué de 3100 dans l'hôpital d'étude entre 2012 et 2014 grâce à ces vaccins.

**Conclusion:**

Suite à l'inclusion des vaccins antipneumococciques conjugués dans le programme de vaccination national, nous avons constaté une forte diminution du nombre d'enfants hospitalisés pour une pneumonie dans l'hôpital d'étude.

## Resumen

**Objetivo:**

Evaluar el impacto de la inmunización con vacunas antineumocócicas conjugadas en todos los casos de hospitalización por neumonía entre niños en Soweto, Sudáfrica.

**Métodos:**

Se utilizaron datos recopilados en el Chris Hani Baragwanath Hospital en Soweto entre 2006 y 2014, es decir, antes y después de abril de 2009, cuando la vacuna antineumocócica conjugada se incluyó por primera vez en el programa de inmunización rutinaria de Sudáfrica. A través de un modelo bayesiano de promedio móvil, generalizado, temporal y autoagresivo y los datos recopilados entre 2006 y 2008, se estimó el número de niños que habrían sido hospitalizados por neumonía entre 2010 y 2014 si no se hubiera utilizado la vacuna antineumocócica conjugada. Estas estimaciones se compararon con los números correspondientes a las hospitalizaciones observadas.

**Resultados:**

Entre 2006 y 2014, 26 778 niños menores de 5 años ingresaron por neumonía en el hospital del estudio, incluyendo 3 388 infectados por el virus de la inmunodeficiencia humana (VIH). Se estimó que, tanto para los niños infectados por el VIH como para el resto, las vacunas antineumocócias conjugadas redujeron el número de hospitalizaciones por neumonía en 2014 en un 33% (intervalo creíble, ICr, del 50%: de 6 a 52) y en un 39% (ICr del 50%: de 24 a 50), respectivamente. En el hospital del estudio entre 2012 y 2014, como resultado de las inmunizaciones con estas vacunas, hubo una reducción de cerca de 3 100 hospitalizaciones por neumonía de niños menores de 5 años.

**Conclusión:**

En nuestro hospital del estudio, después de la introducción de las vacunas antineumocócicas conjugadas en el programa nacional de inmunización, hubo reducciones significativas en las hospitalizaciones por neumonía entre los niños.

## ملخص

**الغرض:**

تقييم أثر التمنيع باستخدام لقاحات المكورات الرئوية على جميع حالات علاج الأطفال بالمستشفيات من الالتهاب الرئوي في سويتو بجنوب أفريقيا.

**الطريقة:**

استخدمنا البيانات التي تم جمعها في مستشفى Chris Hani Baragwanath في سويتو بين عامي 2006 و2014؛ أي قبل وبعد أبريل/نيسان 2009، عندما تم إدراج لقاح المكورات الرئوية لأول مرة في برنامج التمنيع الروتيني في جنوب أفريقيا. وباستخدام نموذج "بايزي" متوسط المجاوزة في الانحدار الذاتي الموسمي والبيانات التي تم جمعها في الفترة ما بين عامي 2006 و2008، فقد قمنا بتقدير عدد الأطفال الذين كان من الممكن دخولهم المستشفى بسبب الالتهاب الرئوي بين عامي 2010 و2014 إذا لم يتم استخدام لقاحات المكورات الرئوية. وتمت مقارنة هذه التقديرات مع الأرقام المقابلة لعمليات الاستشفاء المسجلة في المستشفيات.

**النتائج:**

في الفترة ما بين عامي 2006 و2014، تم إدخال 26778 طفلًا دون الخامسة من العمر – بمن فيهم 3388 طفلاً معروفًا أنهم مصابون بفيروس عوز المناعة البشري (HIV) – إلى المستشفى الخاضعة للدراسة بسبب الالتهاب الرئوي. وتشير تقديراتنا إلى أن لقاحات المكورات الرئوية – بالنسبة للأطفال المصابين بفيروس عوز المناعة البشري (HIV) والأطفال الآخرين – قد قللت عدد حالات الاستشفاء من الالتهاب الرئوي في عام 2014 بنسبة ‏33‏٪ (بهامش موثوقية يبلغ ‏50‏٪، ونسبة ثقة تتراوح ما بين: 6 إلى 52) و‏39‏% (بنسبة ثقة مقدارها ‏50‏%: 24 إلى 50)، على التوالي. في المستشفى الخاضعة للدراسة في الفترة ما بين عامي 2012 و2014، كان هناك تراجع مقداره 3100 حالة في عدد حالات الاستشفاء من الالتهاب الرئوي للأطفال دون سن الخامسة وذلك نتيجة للتطعيمات باستخدام هذه اللقاحات.

**الاستنتاج:**

في المستشفى الخاضعة لدراستنا، وبعد إدخال لقاحات المكورات الرئوية في برنامج التمنيع الوطني، حدثت انخفاضات كبيرة في حالات الاستشفاء من الالتهاب الرئوي بين الأطفال.

## 摘要

**目的:**

为评估接种肺炎球菌结合疫苗对南非索韦托儿童全因肺炎住院治疗的影响。

**方法:**

我们使用 2006 年到 2014 年间——即，在 2009 年四月前后，肺炎球菌结合疫苗首次纳入南非常规免疫计划期间，在索韦托 Chris Hani Baragwanath 医院收集的数据。 我们使用贝叶斯综合季节性自回归滑动平均模型和 2006–2008 年间收集的数据，估算出 2010 年到 2014 年间不使用肺炎球菌结合疫苗的情况下因肺炎住院的儿童人数。 之后将估算值与观察到的相应住院治疗人数进行了比较。

**结果:**

在 2006 年到 2014 年间，26 778 名五岁以下儿童——包括 3388 名已知感染人类免疫缺陷病毒 (HIV) 的儿童——被收入肺炎研究医院接受治疗。 我们估计，在 2014 年，对于已知感染 HIV 的儿童和其他儿童，肺炎球菌结合疫苗分别将肺炎住院治疗人数降低了 33%（50% 置信区间，CrI： 6 至 52）和 39% (50% CrI: 24 至 50）。 在 2012-2014 年间，由于疫苗接种，研究医院五岁以下因肺炎住院治疗的儿童人数预计减少了 3100 人。

**结论:**

在我们的研究医院，在国家免疫计划中引入肺炎球菌结合疫苗之后，儿童肺炎住院治疗人数显著减少。

## Резюме

**Цель:**

Оценить влияние иммунизации пневмококковыми конъюгированными вакцинами на общий показатель госпитализации при пневмонии среди детей в Соуэто, Южная Африка.

**Методы:**

Мы использовали данные, собранные в больнице Криса Хани Барагваната в Соуэто в период с 2006 по 2014 г., то есть до и после апреля 2009 г., когда пневмококковая конъюгированная вакцина впервые была включена в стандартную программу иммунизации в Южной Африке. Используя байесовскую обобщенную сезонную модель авторегрессии скользящего среднего и данные, собранные в 2006–2008 гг., мы подсчитали количество детей, которые были бы госпитализированы с пневмонией в период с 2010 по 2014 г., если бы не использовались пневмококковые конъюгированные вакцины. Затем было проведено сравнение этих оценок с соответствующим количеством госпитализаций.

**Результаты:**

В период с 2006 по 2014 г. 26 778 детей в возрасте до пяти лет, включая 3388 детей, которые, как было известно, были инфицированы вирусом иммунодефицита человека (ВИЧ), были госпитализированы с пневмонией в стационар, участвующий в исследовании. Мы подсчитали, что среди детей, инфицированных ВИЧ, и среди остальных детей пневмококковые конъюгированные вакцины сократили число госпитализаций с пневмонией в 2014 г. на 33% (50% доверительный интервал, ДИ: от 6 до 52) и 39% (50% ДИ: от 24 до 50) соответственно. В стационаре, участвующем в исследовании, в 2012–2014 гг. в результате иммунизации этими вакцинами, по оценкам, насчитывалось на 3100 меньше случаев госпитализации при пневмонии у детей в возрасте младше пяти лет.

**Вывод:**

В нашем стационаре, участвующем в исследовании, после введения пневмококковых конъюгированных вакцин в национальную программу иммунизации наблюдалось значительное сокращение случаев госпитализации при пневмонии среди детей.

## Introduction

Among children younger than five years, *Streptococcus pneumoniae* is the leading cause of bacterial pneumonia and such pneumonia caused an estimated 411 000 deaths in 2010 and 335 000 deaths in 2015 globally.^1^ Although Africa has only 23% of the world’s children younger than five years, it accounts for approximately 43% of the deaths in this age group attributed to bacterial pneumonia.^2^

By June 2016, as part of the fight against bacterial pneumonia, pneumococcal conjugate vaccines were included in many national immunization programmes, including those of 24 low-income and 33 lower-middle-income countries.^3^ The public health impact of seven-, 10- or 13-valent pneumococcal conjugate vaccines against all-cause pneumonia has been investigated in several high-income countries and some middle-income Latin American countries. In these studies, the temporal reduction seen in all-cause pneumonia following childhood immunization with a pneumococcal conjugate vaccine has varied from 0 to 77%.^4^^–^^9^ In Malawi, the impact of a 13-valent vaccine (PCV13) on clinically diagnosed severe or very severe pneumonia has recently been evaluated. In this investigation, however, the study period began after introduction of the vaccine, ran for only 2.5 years and covered a time when only about 50% of the study population were estimated to be receiving all of the scheduled doses of the vaccine.^10^ In addition, there was no attempt to see if impact of the immunizations in Malawi was affected by human immunodeficiency virus (HIV) status. In the present study, we used data collected at a single hospital in the South African township of Soweto over an eight-year period centred on 2009 – the year a pneumococcal conjugate vaccine was first included in South Africa’s routine immunization programme. We used a time-series analysis and a Bayesian model to investigate the apparent impact of infant immunizations with pneumococcal conjugate vaccines on hospitalizations for pneumonia among HIV-uninfected and HIV-infected children younger than five years.

## Methods

### Study setting

In April 2009, a seven-valent vaccine (PCV7; Prevnar®; Wyeth Vaccines, New York, USA) became the first pneumococcal conjugate vaccine to be introduced into the South African public immunization programme.^11^ Only infants beginning their routine childhood vaccinations were offered this vaccine, at six, 14 and 40 weeks of age, and there was no catch-up campaign among older children. In May 2011, however, the seven-valent vaccine was replaced with PCV13 (Prevnar13®; Pfizer Vaccines, Pearl River, USA) and a limited catch-up campaign, for children under 30 months of age, was launched. Although there are limitations in the use of administrative data to estimate vaccine coverage, such data indicate that national coverage with a third dose of pneumococcal conjugate vaccine among children aged nine months of age was 10.4% in 2009, 64.3% in 2010, 89.8% in 2011 and 99.0% in 2012.^12^

### Study facility

The data we analysed came from the Chris Hani Baragwanath Academic Hospital, which was the only public hospital in the South African township of Soweto during our study period. Soweto is a low-income area of the city of Johannesburg, in Guateng province, where unemployment among adults is 53% and 40% of households have a daily income below two United States dollars.^13^^,^^14^ Further detail of Soweto’s socioeconomic status and health-care infrastructure is available from the corresponding author.

### Data source and management

At the study hospital, we established an electronic database covering every patient younger than 14 years who was admitted to a medical ward between 1 January 2006 and 31 December 2014.

#### Cases of pneumonia

Two study physicians categorized the illness in all such patients admitted to the general paediatric ward according to the 10th revision of the International Statistical Classification of Diseases and Related Health Problems (ICD-10).^15^ Patents were considered to have all-cause pneumonia if they had an ICD-10 code of B05.2, B20.6, B25, B59, J10, J12, J12.1, J12.2, J12.8, J12.9, J13, J14, J15, J15.1, J15.2, J15.3, J15.4, J15.5, J15.8, J15.9, J16.8, J17, J18 and/or J18.1. They were considered to have bronchiolitis if coded J21.0, J21.1, J21.8 or J21.9. As the signs and symptoms of pneumonia and sepsis are hard to distinguish in children younger than five years, we considered patients identified as cases of community-acquired neonatal sepsis – i.e. patients given ICD-10 codes of P36.0, P36.1, P36.2, P36.4, P36.8 and/or P36.9 – to be pneumonia admissions.

#### HIV status

Children aged at least 18 months were considered to be HIV-infected and HIV-uninfected if they had been found positive and negative, respectively, when tested using an enzyme-linked immunosorbent assay (ELISA) or polymerase chain reaction (PCR) assay. Children aged at least nine months but younger than 18 months were considered to be HIV-infected if found positive in one PCR assay or two ELISAs and HIV-uninfected if found negative in one ELISA or PCR assay. Younger children were considered HIV-infected and HIV-uninfected if found positive and negative, respectively, in one PCR assay. If a child was hospitalized more than once, an HIV test performed during any one of the hospitalizations was used to assign HIV status to the child – unless the child’s status changed from HIV-uninfected to HIV-infected.

To estimate the age-specific numbers of HIV cases in the whole of Soweto, we used an age pyramid for the township, provided by the Gauteng Department of Health and Social Development,^16^ and estimates of the age-specific prevalences of HIV infection in Gauteng – provided by the Actuarial Society of South Africa’s AIDS and Demographic model.^17^

### Statistics

We used the Gauteng Department of Health and Social Development’s data on the Soweto population (available from the corresponding author) to convert the records of pneumonia admissions at the study hospital to age- and HIV-status-specific annual and monthly incidences of such hospitalizations per 1000 individuals of the same age group and HIV status. To assess the impact of pneumococcal conjugate vaccines on these incidences, we divided our study period into a prevaccine era (2006–2008), a PCV7 era (2010–2011) and a PCV13-era (2012–2014).

By fitting Bayesian generalized seasonal autoregressive integrated moving-average models to the data from the prevaccine era, we estimated what the incidences of all-cause pneumonia hospitalizations might have been, in the PCV7 and PCV13 eras, if there had been no immunizations with any pneumococcal conjugate vaccines.^18^ We created separate models for each HIV status and each of four age groups: under three months; at least three months but under 12 months; at least 12 months but under two years; and at least two years but under five years. Each model included covariates to adjust for the proportion of HIV-infected children on antiretroviral therapy (ART), the influenza season and the number of bronchiolitis-associated admissions and a variable indicating the transition period between PCV7 use and PCV13 use (available from the corresponding author).

For a given period, we calculated the percentage reduction in all-cause pneumonia hospitalizations that probably resulted from immunizations with a pneumococcal conjugate vaccine as: 


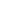
(1)

where ŷ*_t_* is the estimated number of hospitalizations for month *t* that would have occurred in the absence of immunizations with a pneumococcal conjugate vaccine and *y_t_* is the observed number of all-cause pneumonia hospitalizations for month *t*. For our analyses, the prevaccine, PCV7 and PCV13 eras were represented by months 1–36, 49–72 and 73–108, respectively.

The number of hospitalizations prevented was estimated as:


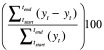
(2)

where T* represents the months when the 25th percentile of the percentage reduction was greater than zero.

For the percentage reductions and numbers of hospitalizations prevented, we report medians and the middle 50% of the simulated distributions – i.e. the 50% credible intervals (CrI). We considered a percentage reduction to be statistically significant if the lower limit of the 50% CrI exceeded zero.

In a sensitivity analysis, we investigated the effect on our main findings of assuming that, in each study year, the prevalence of HIV infection in the children with unknown HIV infection status was that recorded in tests of children of the same age group (available from the corresponding author). In addition, we performed a trend analysis to assess if, over our study period, there had been any temporal changes in bronchiolitis hospitalizations – i.e. changes due to environmental, social or other factors not related to the introduction of pneumococcal conjugate vaccines that may have had similar impacts on pneumonia hospitalizations (available from the corresponding author).

### Ethics

This observational study was approved by the Human Research Ethics Committee of the University of the Witwatersrand, which deemed informed consent unnecessary.

## Results

Between 2006 and 2014 there were 81 791 admissions of children younger than five years at the study hospital. Of these, 26 778 (33%) were coded either as pneumonia (*n* = 23 025) or neonatal sepsis (*n* = 3753) and therefore categorized as pneumonia hospitalizations for our analyses. The 26 778 pneumonia hospitalizations comprised 12 355 (46%) of children confirmed as HIV-uninfected, 3388 (13%) of children confirmed as HIV-infected and 11 035 (41%) of children with unknown HIV infection status. The percentage of pneumonia hospitalizations of children younger than five years represented by children with unknown HIV status decreased from 34% in 2006 to 10% in 2014 (available from the corresponding author).

### Pneumonia hospitalizations

#### Without confirmed HIV infection

**Fig. 1:**
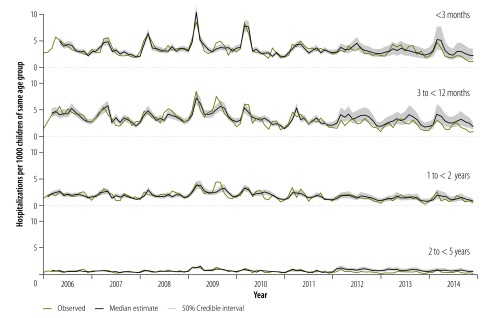
Monthly incidence of pneumonia hospitalizations among children without confirmed positive tests for human immunodeficiency virus, Soweto, South Africa, 2006–2014

Note: The plots show both observed incidence and the modelled median estimates of the incidences that might have been expected if pneumococcal conjugate vaccines had not been used in the public immunization programme. The 50% credible intervals for the estimates are also illustrated.

Among children known to be uninfected with HIV or with unknown HIV status, the observed annual incidences of pneumonia hospitalizations varied little in the pre-PCV era but spiked in 2009 – i.e. the year the first pneumococcal conjugate vaccine was introduced into South Africa’s national immunization programme – and then declined (Table 1). Overall, pneumonia hospitalization incidences in such children – per 1000 children of the same age group – were significantly lower in 2010 than in 2009 and also significantly lower in 2012 than in 2011 (Table 1). The pneumonia hospitalization incidences in the two youngest age groups – which always had higher incidences than the elder age groups – were also significantly lower in 2014 than in 2013 (Table 1; Fig. 1).

**Table 1:** Incidence of observed pneumonia hospitalizations among children younger than five years, Soweto, South Africa, 2006–2014

HIV status, age group	No. of pneumonia hospitalizations per 1000 children of same age group and HIV status (95% CI)								
	2006	2007	2008	2009	2010	2011	2012	2013	2014
**Confirmed infected (n = 3 388)**									
< 3 months	116 (95 to 138)	123 (100 to 145)	113 (91 to 135)	94 (74 to 114)	86 (67 to 105)	54 (39 to 70)	41 (27 to 55)	27 (16 to 38)	37 (24 to 50)
3 to < 12 months	306 (271 to 341)	221 (190 to 251)	247 (214 to 279)	232 (200 to 263)	185 (157 to 214)	79 (61 to 98)	84 (65 to 104)	70 (52 to 88)	56 (39 to 73)
1 to < 2 years	145 (122 to 169)	106 (85 to 126)	84 (65 to 102)	113 (91 to 134)	66 (50 to 83)	53 (38 to 68)	35 (23 to 48)	55 (39 to 71)	40 (26 to 54)
2 to < 5 years	59 (51 to 68)	38 (32 to 45)	35 (29 to 42)	37 (30 to 44)	34 (27 to 40)	23 (17 to 29)	20 (15 to 26)	14 (9 to 19)	15 (10 to 20)
< 5 years	144 (134 to 155)	103 (95 to 112)	103 (94 to 112)	105 (96 to 114)	85 (77 to 94)	51 (44 to 57)	44 (38 to 50)	39 (33 to 45)	36 (30 to 42)
**Confirmed uninfected (n = 12 355)**									
< 3 months	22 (20 to 23)	18 (16 to 20)	31 (29 to 34)	32 (29 to 34)	31 (28 to 33)	30 (28 to 32)	24 (22 to 26)	27 (25 to 29)	19 (17 to 21)
3 to < 12 months	18 (17 to 20)	16 (14 to 18)	19 (17 to 21)	26 (24 to 28)	20 (18 to 22)	21 (19 to 22)	18 (16 to 19)	19 (17 to 21)	13 (12 to 15)
1 to < 2 years	7 (6 to 8)	8 (6 to 9)	10 (9 to 11)	12 (10 to 13)	12 (10 to 13)	10 (9 to 11)	11 (10 to 13)	11 (9 to 12)	9 (8 to 10)
2 to < 5 years	1 (1 to 2)	1 (1 to 2)	2 (2 to 2)	3 (3 to 4)	3 (2 to 3)	3 (3 to 3)	3 (3 to 4)	3 (3 to 3)	2 (2 to 3)
< 5 years	10 (9 to 10)	8 (7 to 8)	12 (12 to 13)	15 (14 to 16)	14 (13 to 14)	14 (13 to 14)	12 (12 to 13)	13 (13 to 14)	10 (9 to 10)
**Not confirmed infected (n = 23 390)^a^**									
< 3 months	43 (40 to 46)	37 (34 to 40)	47 (44 to 50)	51 (48 to 54)	42 (39 to 45)	42 (39 to 44)	36 (33 to 38)	39 (36 to 41)	27 (25 to 30)
3 to < 12 months	47 (44 to 50)	39 (36 to 42)	46 (43 to 49)	59 (55 to 62)	36 (33 to 38)	36 (33 to 38)	29 (26 to 31)	26 (24 to 28)	21 (19 to 23)
1 to < 2 years	24 (22 to 26)	21 (19 to 23)	23 (21 to 26)	32 (30 to 35)	24 (22 to 26)	21 (19 to 23)	17 (15 to 19)	14 (13 to 16)	13 (12 to 15)
2 to < 5 years	7 (6 to 7)	6 (5 to 6)	7 (6 to 7)	11 (10 to 12)	7 (6 to 7)	7 (6 to 7)	6 (5 to 6)	4 (3 to 4)	5 (4 to 5)
< 5 years	26 (25 to 27)	20 (19 to 21)	26 (25 to 27)	33 (32 to 34)	24 (23 to 25)	23 (23 to 24)	20 (19 to 20)	18 (17 to 19)	15 (14 to 16)
**Estimated/known to be infected (n = 6 312)^b^**									
< 3 months	211 (182 to 241)	229 (198 to 260)	160 (134 to 187)	146 (121 to 171)	112 (90 to 134)	75 (57 to 93)	61 (44 to 77)	38 (25 to 51)	52 (36 to 68)
3 to < 12 months	576 (528 to 624)	420 (379 to 462)	473 (428 to 518)	435 (391 to 478)	289 (254 to 325)	129 (105 to 153)	125 (102 to 149)	90 (70 to 111)	80 (60 to 100)
1 to < 2 years	318 (283 to 352)	213 (184 to 242)	170 (143 to 196)	248 (216 to 279)	118 (96 to 140)	100 (79 to 120)	53 (37 to 68)	69 (51 to 87)	59 (42 to 76)
2 to < 5 years	133 (121 to 146)	94 (83 to 105)	85 (74 to 95)	95 (84 to 106)	65 (56 to 74)	44 (36 to 52)	32 (25 to 39)	17 (12 to 22)	27 (20 to 34)
< 5 years	292 (278 to 307)	209 (196 to 221)	201 (188 to 213)	215 (202 to 228)	140 (129 to 151)	87 (78 to 95)	67 (59 to 75)	50 (43 to 57)	55 (47 to 62)
**Estimated/known to be uninfected (n = 20 466)^c^**									
< 3 months	39 (36 to 41)	32 (30 to 35)	45 (42 to 48)	49 (46 to 52)	41 (38 to 43)	41 (38 to 44)	35 (33 to 37)	38 (36 to 41)	27 (25 to 29)
3 to < 12 months	35 (33 to 38)	30 (27 to 32)	36 (33 to 39)	50 (47 to 53)	32 (29 to 34)	34 (31 to 36)	27 (25 to 29)	25 (23 to 27)	20 (18 to 22)
1 to < 2 years	16 (14 to 18)	16 (15 to 18)	20 (18 to 22)	26 (24 to 29)	21 (20 to 23)	19 (17 to 21)	17 (15 to 18)	14 (12 to 15)	13 (11 to 14)
2 to < 5 years	3 (3 to 4)	3 (3 to 4)	5 (4 to 5)	8 (8 to 9)	5 (5 to 6)	6 (5 to 6)	5 (5 to 6)	4 (3 to 4)	4 (4 to 5)
< 5 years	19 (18 to 20)	15 (15 to 16)	21 (21 to 22)	29 (28 to 30)	21 (21 to 22)	22 (21 to 23)	19 (18 to 20)	18 (17 to 18)	15 (14 to 15)
**All (n = 26 778)**									
< 3 months	46 (43 to 49)	39 (36 to 42)	50 (47 to 53)	53 (50 to 56)	44 (41 to 46)	42 (40 to 45)	36 (34 to 38)	38 (36 to 41)	28 (26 to 30)
3 to < 12 months	58 (55 to 62)	44 (41 to 47)	55 (51 to 58)	66 (62 to 69)	42 (39 to 44)	37 (35 to 40)	31 (28 to 33)	27 (25 to 29)	22 (20 to 24)
1 to < 2 years	29 (27 to 31)	23 (21 to 25)	26 (24 to 28)	36 (33 to 38)	25 (23 to 27)	22 (20 to 24)	18 (16 to 19)	15 (14 to 17)	14 (13 to 16)
2 to < 5 years	9 (8 to 10)	7 (6 to 7)	8 (7 to 9)	12 (11 to 13)	8 (7 to 8)	7 (7 to 8)	6 (6 to 7)	4 (4 to 5)	5 (4 to 5)
< 5 years	31 (30 to 32)	24 (23 to 25)	29 (28 to 30)	36 (35 to 37)	26 (25 to 27)	24 (24 to 25)	20 (20 to 21)	19 (18 to 20)	16 (15 to17)

CI: confidence interval; HIV: human immunodeficiency virus.

^a^ Found negative in HIV test or having no available HIV test result.

^b^ The sum of those confirmed to be infected and a proportion – equivalent to the percentage of children in the same year who, when tested for HIV, were found positive – of the hospitalized children with unknown HIV status.

^c^ The sum of those confirmed to be uninfected and a proportion – equivalent to the percentage of children in the same year who, when tested for HIV, were found negative – of the hospitalized children with unknown HIV status.

**Fig. 2:**
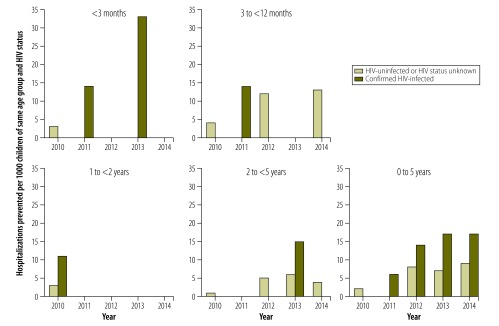
Modelled estimates of the numbers of hospitalizations averted, per 1000 children younger than five years, by the use of pneumococcal conjugate vaccines, Soweto, South Africa, 2010–2014

When we compared the observed data with the results of our modelling, we found that – among the children known to be uninfected with HIV or with unknown HIV status – the observed number of pneumonia hospitalizations in 2010 was 9% (50% CrI: 6 to 12) lower than expected if there had been no immunizations with PCV7. This reduction represents two admissions averted per 1000 children younger than five years who did not have confirmed HIV infection (Table 2; Fig. 2). No such reduction was observed in 2011 (Table 2). However, in each year following the transition to PCV13 in 2011, the immunizations appear to have brought a significant reduction in observed pneumonia hospitalizations. These reductions ranged from 27% (50% CrI: 13 to 39) in 2013 to 39% (50% CrI: 24 to 50) in 2014 (Table 2; Fig. 2). According to our models, in the PCV13 era, immunizations with PCV13 – and/or previous immunizations with PCV7 – averted seven to nine pneumonia hospitalizations per 1000 children younger than five years who did not have confirmed HIV infection (Fig. 2) – i.e. the immunizations averted a total of 2892 (50% CrI: 1522 to 4484) such hospitalizations between 2012 and 2014.

**Table 2:** Estimated change in incidences of pneumonia hospitalization among children younger than five years associated with use of pneumococcal conjugate vaccines in the public immunization programme, Soweto, South Africa, 2010–2014

HIV status, age group	Estimated % change of pneumonia hospitalizations (50% CrI)^ a^				
	2010	2011	2012	2013	2014
**Confirmed infected (n = 3 388)**					
< 3 months	−1 (−15 to 9)	20 (9 to 28)	38 (−3 to 59)	55 (27 to 72)	31 (−16 to 59)
3 to < 12 months	4 (−4 to 11)	15 (5 to 22)	−36 (−132 to 16)	−63 (−185 to 19)	−57 (−159 to 20)
1 to < 2 years	14 (6 to 22)	−2 (−15 to 8)	−20 (−88 to 29)	−73 (−181 to 8)	−15 (−94 to 45)
2 to < 5 years	−1 (−10 to 8)	6 (−3 to 15)	39 (−6 to 59)	51 (3 to 71)	39 (−30 to 67)
< 5 years	4 (−1 to 8)	11 (6 to 16)	24 (2 to 40)	31 (6 to 48)	33 (6 to 52)
**Confirmed uninfected (n = 12 355)**					
< 3 months	10 (4 to 16)	0 (−6 to 6)	17 (−16 to 40)	4 (−43 to 34)	37 (−1 to 59)
3 to < 12 months	8 (3 to 13)	−3 (−9 to 3)	53 (34 to 68)	47 (21 to 65)	62 (41 to 75)
1 to < 2 years	5 (−1 to 10)	2 (−5 to 8)	20 (−10 to 40)	22 (−15 to 45)	38 (1 to 58)
2 to < 5 years	7 (0 to 13)	−1 (−9 to 5)	26 (−2 to 45)	30 (0 to 49)	34 (1 to 54)
< 5 years	9 (5 to 12)	0 (−4 to 3)	38 (25 to 49)	34 (17 to 47)	52 (38 to 63)
**Not confirmed infected (n = 23 390)^b^**					
< 3 months	7 (3 to 12)	−4 (−9 to 0)	12 (−7 to 27)	−5 (−47 to 22)	28 (−12 to 51)
3 to < 12 months	10 (4 to 15)	−1 (−7 to 5)	30 (9 to 46)	27 (−1 to 47)	39 (10 to 57)
1 to < 2 years	10 (4 to 16)	−2 (−10 to 5)	8 (−25 to 31)	11 (−31 to 36)	15 (−37 to 42)
2 to < 5 years	8 (1 to 15)	1 (−6 to 8)	49 (29 to 61)	59 (41 to 70)	45 (17 to 62)
< 5 years	9 (6 to 12)	−2 (−5 to 1)	29 (19 to 38)	27 (13 to 39)	39 (24 to 50)
**Estimated/known to be infected (n = 6 312)^c^**					
< 3 months	6 (−6 to 15)	13 (1 to 23)	46 (9 to 65)	59 (26 to 76)	32 (−28 to 62)
3 to < 12 months	5 (−2 to 12)	13 (4 to 21)	−32 (−123 to 14)	−45 (−196 to 27)	−85 (−232 to 17)
1 to < 2 years	21 (14 to 27)	−5 (−17 to 4)	55 (6 to 74)	14 (−124 to 66)	−5 (−130 to 66)
2 to < 5 years	7 (0 to 14)	5 (−3 to 12)	36 (3 to 54)	55 (5 to 75)	−3 (−142 to 57)
< 5 years	9 (5 to 13)	7 (3 to 12)	37 (20 to 50)	46 (23 to 62)	31 (−6 to 55)
**Estimated/known to be uninfected (n = 20 466)^d^**					
< 3 months	10 (5 to 14)	−4 (−9 to 1)	15 (−7 to 32)	−2 (−39 to 24)	32 (2 to 53)
3 to < 12 months	11 (5 to 16)	−1 (−8 to 5)	33 (10 to 50)	29 (−2 to 50)	41 (12 to 60)
1 to < 2 years	9 (2 to 16)	2 (−6 to 9)	−12 (−54 to 17)	−5 (−57 to 26)	3 (−58 to 35)
2 to < 5 years	11 (2 to 18)	3 (−6 to 11)	49 (25 to 65)	57 (30 to 72)	46 (5 to 67)
< 5 years	10 (7 to 13)	−1 (−4 to 3)	30 (18 to 41)	27 (12 to 40)	41 (26 to 53)

CrI: credible interval; HIV: human immunodeficiency virus.

^a^ Positive values indicate a reduction in pneumonia hospitalizations.

^b^ Found negative in HIV test or having no available HIV test result.

^c^ The sum of those confirmed to be infected and a proportion – equivalent to the percentage of children in the same year who, when tested for HIV, were found positive – of the hospitalized children with unknown HIV status.

^d.^The sum of those confirmed to be uninfected and a proportion – equivalent to the percentage of children in the same year who, when tested for HIV, were found negative – of the hospitalized children with unknown HIV status.

HIV: human immunodeficiency virus.

Note: The numbers were estimated in models based on the assumption that, if pneumococcal conjugate vaccines had not been used in the public immunization programme, the trends seen in hospitalization before the vaccines were introduced would continue. The plots show the values for the four age groups studied and for all of these groups combined.

In 2010, according to our models, children known to be uninfected with HIV or with unknown HIV status in all five of the age groups we investigated had significantly lower incidences of pneumonia hospitalization than might have been expected had there been no immunizations. In that year, in each age group, such immunizations appear to have averted one to four admissions per 1000 children in the same age group who did not have confirmed HIV infection (Table 2; Fig. 2). Between 2011 and 2014, however, there were no corresponding significant reductions in two of the age groups – i.e. among children younger than three months or aged at least one year but younger two years. Significant reductions were observed in both 2012 (30%; 50% CrI: 9 to 46) and 2014 (39%; 50% CrI: 10 to 57) among the children who were aged at least three months but under 12 months and also in 2012, 2013 and 2014 (45−59%) for the eldest age group we considered (Table 2). In 2012 and 2014, the immunizations appear to have averted 12 (50% CrI: 3 to 24) and 13 (50% CrI: 2 to 28) pneumonia hospitalizations of children aged at least three months but under 12 months per 1000 children of the same age group who did not have confirmed HIV infection, respectively (Fig. 2). Among the children we investigated who were aged at least two years, the number of pneumonia hospitalizations averted during the whole PCV13 era was four to six per 1000 children of the same age group who did not have confirmed HIV infection (Fig. 2).

When we allocated the children with unknown HIV status to the HIV-uninfected or HIV-infected groups – according to the corresponding prevalences of HIV infection recorded among tested children – we obtained a similar point estimate for the overall reduction in the incidence of pneumonia hospitalization among HIV-uninfected children younger than five years between 2010 and 2014: 41% (50% CrI: 26 to 53). When stratified by age group, the estimated reductions were also generally similar to those observed in the primary analysis (Table 2).

#### With confirmed HIV infection

**Fig. 3:**
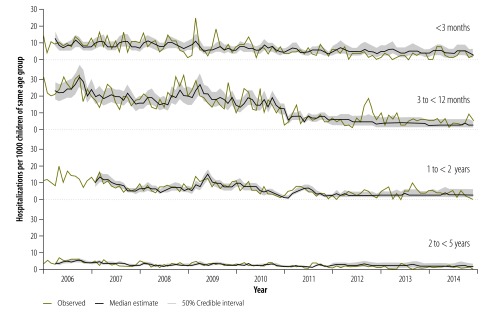
Monthly incidence of pneumonia hospitalizations among children found positive in tests for human immunodeficiency virus, Soweto, South Africa, 2006–2014

Note: The plots show both observed incidence and the modelled median estimates of the incidences that might have been expected if pneumococcal conjugate vaccines had not been used in the public immunization programme. The 50% credible intervals for the estimates are also illustrated.

Among children with known HIV infection, the highest incidence of all-cause pneumonia hospitalization in the prevaccine era was recorded among the children aged at least three months but younger than 12 months, followed by those younger than three months (Table 1). The incidence of pneumonia hospitalization among children with known HIV infection decreased during the prevaccine period, increased in 2009 – in all age groups – and then declined again (Table 1; Fig. 3). In the prevaccine (117 versus 23; *P* < 0·001), PCV7 (68 versus 23; *P* < 0·001) and PCV13 eras (40 versus 18; *P* < 0·001), the incidence of all-cause pneumonia hospitalization per 1000 was 2- to 5-fold higher in the children with known HIV infection than in the other children (available from the corresponding author).

In the children with known HIV infection, the annual incidence of pneumonia hospitalizations declined across the prevaccine era (Table 1; Fig. 3). The incidence of pneumonia hospitalization observed among such children in 2010 was similar to the modelled estimate – i.e. the value that might have been expected if there had been no immunizations with pneumococcal vaccine conjugates. The corresponding incidences observed in 2011 and 2014 were 11% (50% CrI: 6 to 16) and 33% (50% CrI: 6 to 52) lower than the corresponding modelled estimates. During the whole PCV13 era, such reductions represented the equivalent of 14 to 17 pneumonia hospitalizations averted per 1000 children younger than five years with known HIV infection (Fig. 2).

In terms of the post-2009 reductions in the incidence of pneumonia hospitalizations – among children with known HIV infection – that we tentatively attributed to PCV7 and/or PCV13, the five age groups we investigated gave varied results. Among children younger than three months, the observed incidence of pneumonia hospitalizations was significantly lower than the modelled estimate in both 2011 (20%; 50% CrI: 9 to 28) and 2013 (55%; 50% CrI: 27 to 72). Other significant reductions were recorded only in 2011 for the children aged at least three months but under 12 months (15%; 50% CrI: 5 to 22) and in 2013 for the children aged at least two years but less than five years (51%; 50% CrI: 3 to 71; Table 2).

In the sensitivity analysis, where some of the children with unknown HIV status were assumed to be HIV-infected, there was no consistent significant reduction in any age group from 2010 onwards (Table 2). Although the median of the difference between the overall observed incidence of pneumonia hospitalization in 2013 and the corresponding modelled estimate was 46% (50% CrI: 23 to 62) – mainly due to a relatively low incidence among the children aged at least two years but less than five years (Table 2) – there was no such significant difference recorded for 2014.

### Bronchiolitis hospitalizations

In children without known HIV infection from four of our study age groups, the annual incidence of bronchiolitis hospitalization did not change significantly across our study period (χ^2^ test for trend; *P* > 0·05). The only children to show significant changes over time were those aged at least three months but younger than 12 months (available from the corresponding author). However, the incidences of bronchiolitis hospitalization observed in the PCV7 and PCV13 eras were still higher than the modelled estimates based on the incidences observed in the prevaccine era (available from the corresponding author).

## Discussion

The introduction of PCV7 followed by PCV13 into the South African public immunization programme appears to be temporally associated with a reduction in all-cause pneumonia hospitalization of children younger than five years. We used mathematical models to estimate the number of pneumonia hospitalizations that might have been expected in the absence of any immunizations with pneumococcal conjugate vaccines – for those years when such immunizations occurred. These models indicated that, in Soweto in 2014, as a result of the introduction of PCV7 and/or PCV13, there were 39% fewer pneumonia hospitalizations of children who were not known to be HIV-infected. The immunizations appear to have averted an estimated 1116 (50% CrI: 559 to 1798) hospitalizations among Soweto children without confirmed HIV infection in 2014 alone. The corresponding values for children known to be HIV-infected were lower: a 33% (50% CrI: 6 to 52) reduction in the incidence of pneumonia hospitalizations resulting in 68 (50% CrI: 9 to 153) pneumonia hospitalizations being averted in Soweto.

Our findings for children without known HIV infection are consistent with observations from similar studies from Australia – which reported a corresponding reduction of 17 to 29% in children aged at least two years but younger than five years^19^ – and the USA – which reported a reduction in all-cause pneumonia hospitalization in children younger than two years of about 39%.^20^ However, the hospitalizations investigated in these studies all occurred after the introduction of PCV7 into public immunization programmes. In the present study the reduction in all-cause pneumonia hospitalization observed among children without known HIV infection was relatively small while PCV7 was being used and only became significant following the implementation of PCV13.

We estimated that, as the result of immunizations with PCV7 or, more likely, with PCV13, in Soweto between 2012 and 2014 – i.e. after PCV7 had been replaced with PCV13 – there had been a 32% decline in the incidence of all-cause pneumonia hospitalization among children younger than five years without known HIV infection. This decline exceeds the 7% reduction observed for lower respiratory tract infection in African efficacy trials of a nine-valent PCV9 – which included serotypes 1 and 5 but not the serotypes 3, 6A, 7F and 19A contained in the PCV13 product – and the reductions recorded with other pneumococcal conjugate vaccines elsewhere.^4^^,^^20^^–^^25^ Given the strength of its antibody responses to serotype 19F and its efficacy against the nasopharyngeal acquisition of this serotype,^26^ PCV13 might have higher effectiveness against serotype 19F pneumonia than PCV7 or PCV9. A post-hoc analysis of data from the PCV9 efficacy trial in South Africa indicated that the vaccine had caused a 17% (95% CI: 7 to 26) reduction in all-cause clinical pneumonia hospitalization – the definition of which excluded bronchiolitis cases.^27^ We used trends in the incidence of bronchiolitis hospitalization to investigate if the changes we observed, in the incidence of pneumonia hospitalization, might have been attributable to differences in infection with respiratory syncytial virus or epidemics of infection with other respiratory viruses during the study period. We also used bronchiolitis hospitalizations as an indirect measure of any temporal changes in hospitalization patterns for lower respiratory tract infection. The incidence of bronchiolitis hospitalization in the PCV13 era was similar to that in the prevaccine PCV except for a small reduction in the incidence among children aged at least three years but younger than 12 months – which was less than the decline observed in the incidence of pneumonia hospitalization in this age group.

It is unclear why the apparent beneficial impact that we observed for PCV7 and/or PCV13 against pneumonia was greater than that observed in PCV9 efficacy trials. However, it seems possible that the difference reflects the indirect protection offered by pneumococcal conjugate vaccines when they are used in general immunization programmes. After most children in a population have received such vaccines, both the vaccinated and unvaccinated have a lower risk of pneumococcal disease as a result of their reduced exposure to circulating vaccine serotypes. In Soweto there is biological evidence of such indirect benefits, at least for PCV13: a 68% reduction in PCV13-serotype colonization among children between 2010 and 2012 and a corresponding 66% reduction in vaccine serotype colonization among unvaccinated adults.^28^ The prevalence of PCV13 serotype colonization is likely to have declined further between 2012 and 2014 with the ongoing use of PCV13.^29^ The significant reduction we observed in pneumonia hospitalizations among infants younger than three months provides further evidence of the indirect benefits of PCV7 and/or PCV13 to Soweto – since such children should have received no doses or only one dose of pneumococcal conjugate vaccine. In South Africa, immunization with PCV7 and/or PCV13 also seemed to have indirect benefits when tested against invasive pneumococcal disease in adults and infants younger than 10 weeks.^30^

The results of the present study indicate that, in Soweto at least, the additional serotypes included in PCV13 make this vaccine more effective against childhood bacterial pneumonia than PCV7. Immunizations with PCV7 only seem to have reduced pneumonia hospitalizations by less than 10%, if at all, in 2010 and 2011 but the reductions seen every year in the PCV13 era were considerably greater. In Israel, the incidence of hospitalization of children younger than five years because of community-acquired alveolar pneumonia was only reduced after PCV7 was replaced with PCV13.^31^ Despite the greater apparent impact shown in our study compared with those seen in the PCV9 efficacy trials, we refrain from a detailed comparison of our results, which are based on so-called 2+1 schedule – i.e. of two primary doses plus a booster – with those of the efficacy trials based on a so-called 3+0 schedule – i.e. of three primary doses without a booster. A systematic review has indicated that, when used for routine immunizations against pneumonia in high-income countries, the 2+1 and 3+0 schedules have similar effectiveness. The immunizations we investigated probably offered both direct and indirect protection whereas the PCV9 efficacy trials primarily measured direct protection.^5^

The interpretation of time-series analyses on the impact of pneumococcal conjugate vaccines against pneumonia in HIV-infected children is complicated by potential covariates and confounders. After having accounted for temporal changes in ART coverage among children with known HIV infection, we observed a 30% lower incidence of all-cause pneumonia hospitalization in HIV-infected children younger than five years in 2014, compared with the modelled estimates. We did not, however, demonstrate any significant age-group-specific reductions in such incidence between consecutive years – perhaps because the incidence of pneumonia hospitalization among HIV-infected children was already declining in the prevaccine era because of increased coverage of ART and cotrimoxazole prophylaxis. It also seems possible that HIV-infected children have a less robust immune response to pneumococcal conjugate vaccines and are, in consequence, less protected against pneumonia by such vaccines.^22^^,^^32^^,^^33^

Our findings appear to be the first from an analysis – of the temporal association of pneumonia hospitalizations with the introduction of pneumococcal conjugate vaccines into the public immunization programme of any African country – that includes prevaccine data. Our findings add to the increasing body of evidence indicating that routine immunization with pneumococcal conjugate vaccines prevents a range of disease outcomes when used on different schedules and in areas with high- or low-disease burdens.
